# Postbiotic-based recombinant receptor activator of NF-κB ligand enhanced oral vaccine efficiency in chicken

**DOI:** 10.1007/s00253-024-13237-9

**Published:** 2024-06-26

**Authors:** Biao Xuan, Jongbin Park, Seojin Choi, Eun Bae Kim

**Affiliations:** 1 Department of Animal Science, College of Agriculture, Yanbian University, Yanji, 133002 China; 2 Korea Research Institute of Bioscience and Biotechnology (KRIBB), Microbiome Convergence Research Center, Daejeon, 34141 South Korea; 3 Department of Applied Animal Science, College of Animal Life Sciences, Kangwon National University, Kangwon-Do, Chuncheon, 24341 Republic of Korea

**Keywords:** Postbiotics, Recombinant protein, RANKL, Oral vaccine, Chicken

## Abstract

**Abstract:**

Functional M cells are differentiated by receptor activator of NF-κB ligand (RANKL) and capture of luminal antigens to initiate immune responses. We aimed to use postbiotic-based recombinant chicken RANKL (cRANKL) to promote M cell differentiation and test the efficacy of oral vaccines. Chicks were divided into three groups that were administered phosphate-buffered saline (PBS), cell extracts of wild-type *Lactococcus lactis* subsp. *lactis* IL1403 (WT_CE), or cell extracts of recombinant *L. lactis* expressing cRANKL (cRANKL_CE). The expression of the M cell marker was measured, and the gut microbiome was profiled. The efficiency of the infectious bursal disease (IBD) vaccine was tested after 12 consecutive days of administering cRANKL_CE. The chickens that were administered cRANKL_CE (*p* = 0.038) had significantly higher Annexin A5 (*ANXA5*) mRNA expression levels than those in the PBS group (PBS vs. WT_CE, *p* = 0.657). In the gut microbiome analysis, no significant changes were observed. However, the relative abundance of *Escherichia-Shigella* was negatively correlated (*r* =  − 0.43, *p* = 0.019) with *ANXA5* mRNA expression in Peyer’s patches. cRANKL_CE/IBD (*p* = 0.018) had significantly higher IBD-specific faecal IgA levels than PBS/IBD (PBS/IBD vs. WT_CE/IBD, *p* = 0.217). Postbiotic-based recombinant cRANKL effectively improved the expression of M cell markers and the efficiency of oral vaccines. No significant changes were observed in the gut microbiome after administration of postbiotic-based recombinant cRANKL. This strategy can be used for the development of feed additives and adjuvants.

**Key points:**

• *Postbiotic-based recombinant cRANKL enhanced the expression of ANXA5 in chicken.*

• *The relative abundance of Escherichia-Shigella was negatively correlated with ANXA5 expression.*

• *Postbiotic-based recombinant cRANKL effectively improved the efficiency of oral vaccine.*

**Supplementary Information:**

The online version contains supplementary material available at 10.1007/s00253-024-13237-9.

## Introduction

The International Scientific Association for Probiotics and Prebiotics (ISAPP) consensus defines postbiotics as “preparations of inanimate microorganisms and/or their components that confer a health benefit on the host”. It includes components such as cell wall fragments, metabolites, and other microbial derivatives (Salminen et al. 2021). Postbiotics contain a wide range of molecules, including peptidoglycans, surface proteins, cell wall polysaccharides, secreted proteins, bacteriocins, and organic acids, which have positive effects on the host, such as anti-inflammatory, immunomodulatory, antitumor, antimicrobial, and antioxidant effects (Nataraj et al. 2020; Teame et al. 2020). However, most studies have used wild-type postbiotics, which exhibit limitations with respect to the regulation of specific pathways.

Microfold (M) cells are specialised immune cells located in the gut-associated lymphoid tissue (GALT) and play an essential role in the initiation of the intestinal immune response that transports luminal antigens via the intestine toward GALT (Foussat et al. 2001). Receptor activator of NF-κB ligand (RANKL) can induce differentiation of functional M cells (Kunisawa et al. 2008; Knoop et al. 2009; Kanaya et al. 2012). Knoop et al. showed that purified recombinant mouse RANKL produced by recombinant *E. coli* differentiated M cells in the small intestine of RANKL-null mice (Knoop et al. 2009). In addition, Kim et al. showed that oral administration of recombinant *L. lactis*-secreting mouse RANKL significantly increased the number of mature M cells in the mouse small intestine (Kim et al. 2015). These studies demonstrate that recombinant RANKL produced by bacteria exhibits bioactivity. Moreover, owing to the characteristics of M cells, the promotion of M cell differentiation may be a viable strategy to increase the efficiency of oral vaccines (Yamamoto et al. 2012). Therefore, the use of postbiotic-based recombinant RANKL not only has the potential to increase the activity of M cells but also may demonstrate a synergistic effect of recombinant protein and postbiotics. Moreover, postbiotics influence the changes in the gut microbiome (Ozma et al. 2022). Therefore, it is necessary to determine the influence of postbiotic-based recombinant proteins on the gut microbiome.

In this study, the bioactivity of the postbiotic-based recombinant cRANKL was tested by measuring the expression of M cell markers in chickens, and changes in the intestinal microbial community were profiled. Therefore, we investigated the possibility of using postbiotic-based recombinant cRANKL as an adjuvant or additive.

## Materials and methods

### Microorganism strains and growth conditions

Wild-type *Lactococcus lactis* subsp. *lactis* IL1403 (Simon and Chopin 1988, strain collected from Seoul National University) was used as the host bacterium for target protein expression. Wild-type and recombinant strains were grown in M17 medium (MBcell, Korea) supplemented with 5 g/L of glucose (M17G) without antibiotics or with erythromycin (5 μg/mL) and chloramphenicol (5 μg/mL) at 30 °C, respectively.

### Gene synthesis and plasmid construction

**Fig. 1:**
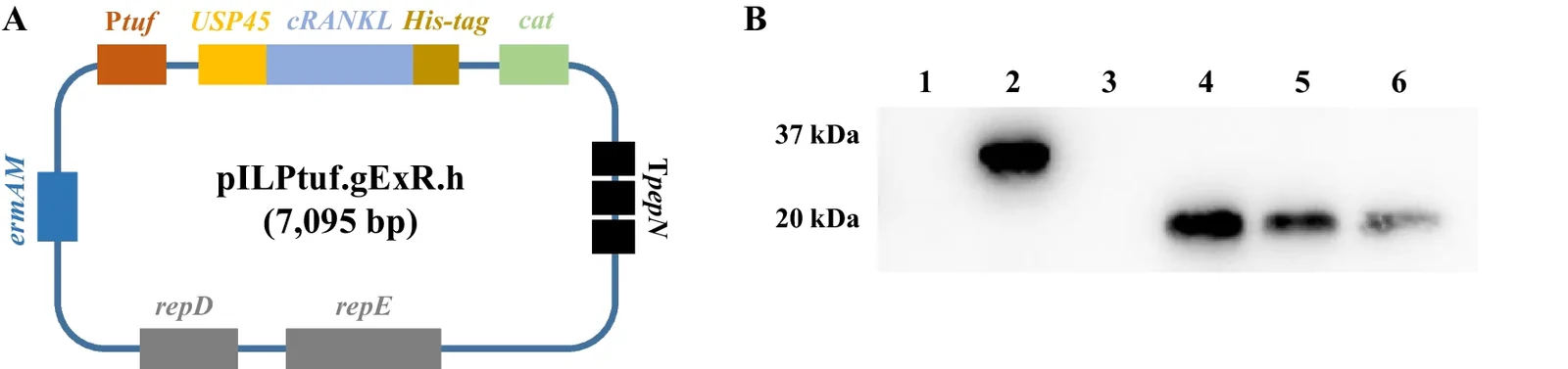
Production and secretion of recombinant cRANKL from recombinant *L. lactis*. **A** Schematic diagram for construction of pILPtuf.gExR.h vector. **B** Western blot for detecting recombinant cRANKL from cell extracts and cell-free culture supernatant. Lane 1, cell extracts of *L. lactis* IL1403; Lane 2, cell extracts of *L. lactis* IL1403 (pILPtuf.gExR.h); Lane 3, cell-free culture supernatant of *L. lactis* IL1403 (pILPtuf.gExR.h); Lanes 4–6: commercial His-tagged calmodulin (18 kDa) 1.5, 1, and 0.5 μg, respectively

Two cRANKL amino acid (aa) sequences were obtained from the National Center for Biotechnology Information (NCBI): 400 aa (NCBI accession number: NP_001076830.1) and 318 aa (NCBI accession number: CDZ92724.1). These two proteins had the same extracellular domain sequences. Based on the results of alignment with the soluble form of mouse RANKL (mRANKL) (Knoop et al. 2009; Kim et al. 2015), the one of candidate gene fragments was selected, and the extracellular form of cRANKL was obtained from Sutton et al. (2015). To secrete the target protein from recombinant *L. lactis*, the signal peptide of USP45 (van Asseldonk et al. 1993) was added to the N-terminus of the target gene. The His-tag (His6x) was added to the C-terminus of the target gene to detect the target proteins. The restriction sites of *Nde*I and *Xho*I were located at the two ends for insertion and vector ligation. The vector construction is shown in Fig. 1A. The designed amino acid sequences were codon-optimised using DNAWorks v3.2.4 (Hoover and Lubkowski 2002) based on the *L. lactis* IL1403 codon usage table, and primers were used to synthesise the insert sequences using the overlap PCR method. The plasmid DNA pILPtuf.Mb (Kim et al. 2009) vector was used as the backbone. The insert and vector were ligated at *Nde*I and *Xho*I restriction sites and transformed into wild-type *L. lactis* IL1403 competent cells. All insert sequences are shown in Fig. S1.

### SDS-PAGE and western blot assay

Wild-type and recombinant *L. lactis* were cultured in 10 mL of M17G medium without antibiotics or with antibiotics at 30 °C for 10 h, respectively. For the preparation of cell extracts, 10 mL of cultured cells was harvested by centrifugation 3,134 × *g* for 10 min at 4 °C, and cell pellets were washed by sterilised distilled water twice and resuspended in 200 µL of sterilised 1 × PBS. Subsequently, the solution containing the cell pellet was supplemented with 0.5 g of sterilised glass beads (0.5 mm), followed by disruption using a taco Prep Bead Beater (GeneReach, Taiwan) for 39 s. Cell debris was removed from the cell extracts by centrifugation at 12,300 × *g* for 5 min at 4 °C. For the preparation of secreted proteins, after centrifugation at 3134 × *g* for 10 min at 4 °C, the culture supernatant was filtered using the 0.2-µm filter (Minisart® Syringe Filter, Sartorius) and then precipitated with trichloroacetic acid (TCA, 4:1) at 4 °C for 1 h. The precipitates were washed twice with 1 mL of pre-chilled acetone, and centrifugation was performed at 12,300 × *g* for 5 min at 4 °C. The precipitated pellets were dried at 65 °C for 10 min and dissolved in 200 µL of sterilised 1 × PBS. To quantify the amount of target protein produced, commercial recombinant His-tagged human calmodulin (MERCK, Darmstadt, Germany) protein (18 kDa) was used for the construction of the standard curve with concentrations of 0.5, 1, and 1.5 µg.

The proteins from the total cell extracts or cell-free culture supernatant were separated by SDS-PAGE and transferred onto a nitrocellulose membrane (2 μm; Bio-Rad, Germany). The membrane was blocked with 2.5% (w/v) skim milk in 1 × tris-buffered saline (TBST, 0.1% Tween® 20 detergent) at 25 °C for 1 h. After blocking, the membrane was washed with 1 × TBST thrice for 10 min per wash and incubated with anti-His6x monoclonal antibody (1:500, R&D Systems, USA) at 4 °C for 12 h with shaking. After washing thrice with 1 × TBST, the membrane was visualised with ECL reagents (Bio-Rad, USA).

### Validation of the bioactivity of recombinant cRANKL in vivo

**Fig. 2:**
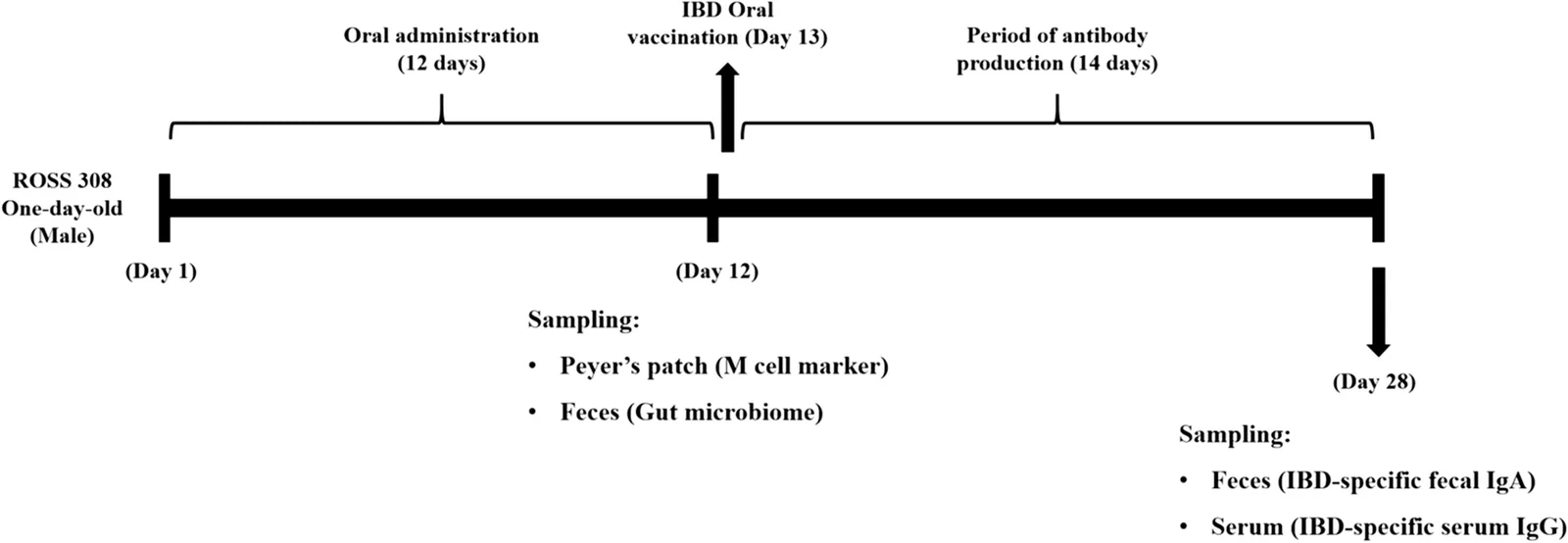
Schematic view of postbiotic-based recombinant RANKL administration, and oral immunization with infectious bursal disease (IBD) vaccine

One-day-old male ROSS 308 chicks were randomly assigned to three groups, namely PBS, WT_CE, and cRANKL_CE, with ten chicks in each group. The chicks in the PBS group were administered sterilised 1 × PBS. WT_CE and cRANKL_CE groups were administered cell extracts from wild-type *L. lactis* IL1403 and the recombinant strain *L. lactis* IL1403 (pILPtuf.gExR.h), respectively. For the preparation of cell extracts, 12-h-cultured wild-type or recombinant strain was harvested by centrifugation 3134 × g for 10 min at 4 °C, and cell pellets were washed by sterilised distilled water twice and resuspended in sterilised 1 × PBS. Subsequently, the solution containing the cell pellet was supplemented with 0.5 g of sterilised glass beads (0.5 mm), followed by disruption using a taco Prep Bead Beater (GeneReach, Taiwan) for 39 s. Glass beads were removed from the cell extracts by centrifugation at 12,300 × g for 5 min at 4 °C. Then, the extracted cell extracts were inoculated in M17G medium and found to contain no viable bacteria. The prepared cell extracts were administered orally to each chick using a 1 mL syringe without a needle. The dosage of cell extracts is shown in Table 1. All groups were fed for 12 consecutive days and sampled on the 13th day (Table 1, Fig. 2).

**Table 1:** Daily oral dosage of cell extracts in chicken experiment

Day	Age (days)	ROSS 308 (weight, g)	cRANKL_CE^1^		WT_CE^1^
			× mL CE/chicken	cRANKL (µg/chicken)	× mL CE/chicken
1	1	40	6.7	14.74	6.7
2	2	60	10	22	10
3	3	78	13	28.6	13
4	4	98	16.5	36.3	16.5
5	5	121	20.5	45.1	20.5
6	6	147	25	55	25
7	7	175	29.5	64.9	29.5
8	8	207	35	77	35
9	9	242	41	90.2	41
10	10	281	47.5	104.5	47.5
11	11	323	54	118.8	54
12	12	369	62	136.4	62

^1^*CE*, cell extracts

Yield of cRANKL 2.2 µg/mL

Peyer’s patches of ileum samples were extracted to determine the expression level of the M cell marker by measuring *ANXA5* mRNA expression. Total RNA was extracted using TRIzol® (Thermo Fisher, Korea) according to the manufacturer’s instructions, and cDNA was synthesised using the PrimeScriptTM RT reagent Kit (Takara Bio, Japan). Quantitative real-time PCR (qRT-PCR) was conducted using TB Green® Premix Ex Taq™ (Tli RNaseH Plus, TAKARA, Japan) with specific primers ANXA5-F:5′-AGTATACAAGAGGCACCGTG-3′; ANXA5-R:5′-GTCTCATCAAAGATACCATC-3′, and primers for the housekeeping gene GAPDH-F:5′-GTGGTGCTAAGCGTGTTATCATC-3′; GAPDH-R:5′-GGCAGCACCTCTGCCATC-3′ (Olias et al. 2014). The mRNA level was presented as 2^−ΔCt^, where Ct, threshold cycle for target amplification, and ΔCt, Ct_taget gene_ (specific genes for each sample) − Ct_internal reference_ (housekeeping gene for each sample).

### 16S rRNA amplicon sequencing

On the 13th day, genomic DNA was extracted from faecal samples using a NucleoSpin Soil kit (Macherey–Nagel, Düren, Germany), according to the manufacturer’s instructions. DNA samples (5 ng) were used to amplify the 16S ribosomal RNA V4 region using Takara Ex-Taq DNA polymerase (Takara Bio) with universal primer sets (forward:5′-GGACTACHVGGGTWTCTAAT-3′ and reverse:5′-GTGCCAGCMGCCGCGGTAA-3′) (Han et al. 2018). After amplification, all samples were normalised to 50 ng per sample. A DNA library was constructed and sequenced using the Illumina MiSeq platform (Illumina, San Diego, CA, USA), generating 2 × 300 bp paired-end reads.

### Bioinformatic analysis of the gut microbiome

To analyse the microbial community, de-multiplexed and pre-processed sequence reads were imported into Quantitative Insights Into Microbial Ecology (QIIME2, version 2021.2) (Bolyen et al. 2019). Barcode and primer removal, quality control, amplicon sequence data correction, and de-replication were performed using the DADA2 software package (Callahan et al. 2016). Sequence reads were truncated to 200 bp using an in-house Perl script. Feature tables and representative sequence files were merged for downstream analysis using QIIME2. Taxonomic classification was assigned using the SILVA 132 database, with 99% identity based on the V4 16S region. All classifications were performed within QIIME2 and were assigned using the naïve Bayesian algorithm available in the Sklearn Python library. For phylogenetic diversity analysis, alpha and beta diversities were calculated using the q2-diversity plugin and included Faith’s phylogenetic diversity and weighted and unweighted UniFrac distances. Differential abundance analysis of microbiota was performed using an in-house Perl script. The relationship between relative abundance and *ANXA5* mRNA expression was assessed by Pearson’s correlation efficient (*r*) and *p* values from simple linear regression. Statistical significance was set at *p* < 0.05.

### Serum calcium assay

The serum calcium concentration was measured using a Calcium Colorimetric Assay Kit (BioVision Inc., USA). All serum samples were diluted to ten-folds (10 μL of serum and 90 μL of Chromogenic Reagent), and 60 μL of Calcium Assay Buffer was added to each well and mixed gently. The samples were incubated for 10 min at room temperature in the dark. After incubation, the optical density (OD) was analysed by measuring the absorbance of the samples at 575 nm.

### Evaluation of oral vaccine efficiency

After 12 consecutive days of oral administration of postbiotic-based recombinant cRANKL, chickens were orally immunised with the infectious bursal disease (IBD) vaccine on the 13th day. The experimental schedule is shown in Fig. 2. The IBD vaccine (PoulShot® Gumboro) was purchased from the Central Vaccine Research Institute, Korea. The dosage of oral vaccination was based on vaccine programme guidelines. The relationship between faecal IgA and serum IgG was assessed by Pearson’s correlation efficient (*r*) and *p* values from simple linear regression. Statistical significance was set at *p* < 0.05.

### Statistical analysis

Statistical analysis was performed using an in-house Perl script and R (v4.1.4). For significance analysis, the Kruskal–Wallis test was performed followed by Dunn’s posthoc test, and the data are expressed as follows: *p < 0.05, **p < 0.01, ***p < 0.001.

## Results

### Construction of recombinant strains

Although the correct insert sequences were cloned into *L. lactis*, some transformants did not form colonies, or mutations were found after sequencing (Figure S2). To confirm the expression of cRANKL, intracellular and secreted proteins were precipitated and analysed by western blotting. cRANKL was not detected in cell extracts of the wild-type strain. In the recombinant strain, only intracellular cRANKL was detected, with an expected size of 31.74 kDa (Fig. 1B). According to the standard curve, intracellular production of cRANKL was 2.2 μg/mL.

### Validation of bioactivity of postbiotic-based recombinant cRANKL in vivo

**Fig. 3:**
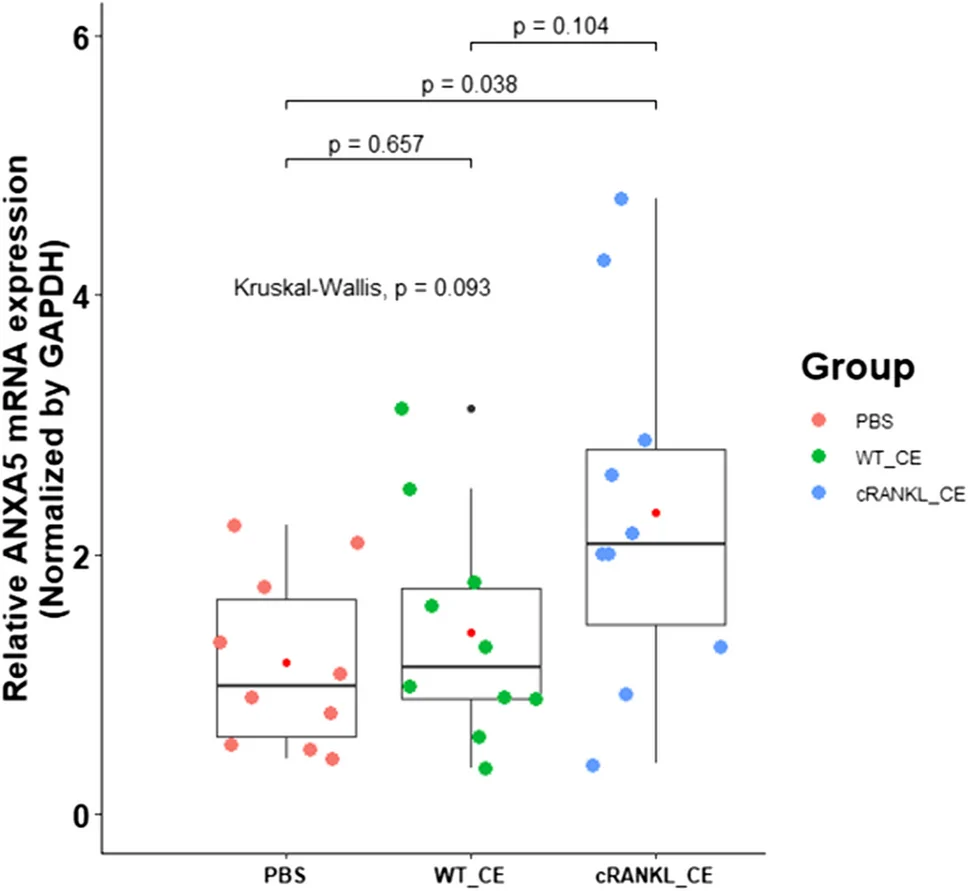
qRT-PCR analysis of ANXA5 expression to validate the bioactivity of postbiotic-based recombinant cRANKL from cell extracts in chicken Peyer’s patch. For significance tests, Kruskal–Wallis test followed by Dunn’s post-hoc test. Mean, red point; median, black line

To validate the bioactivity of postbiotic-based recombinant RANKL from *L. lactis* cell extracts in ROSS 308 chickens, RT-PCR was performed to detect ANXA5-high M cells from the Peyer’s patches of chickens. As shown in Fig. 3, compared with the PBS group, the cRANKL_CE (*p* = 0.038) group showed significantly higher *ANXA5* mRNA expression levels, and no significant difference was observed between the PBS and WT_CE groups (*p* = 0.657). Despite the absence of significant differences between the WT_CE and cRANKL_CE groups, the cRANKL_CE group had higher *ANXA5* expression levels than the WT_CE group (*p* = 0.104). These results indicate that cRANKL from cell extracts of recombinant *L. lactis* affected *ANXA5* expression in M cells. No significant differences were observed in body weight gain between the groups (Figure S3).

### Gut microbial diversity

To compare the gut microbial diversity and communities, the alpha and beta diversities of the PBS, WT_CE, and cRANKL_CE groups were investigated from normalised microbiome sequencing reads. For alpha diversity, four indices that observed features, Evenness, Shannon index, and Faith’s phylogenetic diversity (Faith PD), were measured. No significant difference was observed in the observed features and Faith PD. However, the Evenness and Shannon indices of cRANKL_CE were significantly higher than those of the WT_CE group (Figure S4). For beta diversity, principal coordinate analysis (PCoA) of the unweighted and weighted UniFrac distances was performed. No significant differences were observed among the three groups (Figure S5).

### Gut microbial composition and its correlation with the expression of ANXA5

To compare the differences in the major gut microbial taxa between the PBS, WT_CE, and cRANKL_CE groups, the microbial composition in these three groups was examined. The overall microbial composition in the gut was not significantly different among the three groups (Tables 1 and S1). However, at the phylum level, *Firmicutes* in the cRANKL_CE group were more abundant than those in the PBS (*p* = 0.058) and WT_CE groups (*p* = 0.244). At the genus level, *Lactobacillus*, *Terrisporobacter*, *Enterococcus*, *Turicibacter*, *Staphylococcus*, *Tuzzerella*, and *Pseudomonas* were significantly different. Compared to the WT_CE group, the abundance of *Escherichia-Shigella* decreased in the cRANKL_CE group (*p* = 0.062). Compared to the PBS group, the abundance of *Lysinibacillus* decreased in both the WT_CE (*p* = 0.070) and cRANKL_CE (*p* = 0.163) groups. Moreover, no significant differences were observed among the three groups with respect to the genus *Lactococcus* (Table 1). In correlation analysis, the relative abundance of *Escherichia-Shigella* was negatively correlated (*r* =  − 0.43, *p* = 0.019) with host *ANXA5* mRNA expression in Peyer’s patches (Fig. 5).

### Serum calcium assay

To detect changes in serum calcium concentration attributed to bone metabolism disorder after the administration of recombinant cRANKL, serum calcium levels were measured. The results showed no significant difference in serum calcium levels among the three groups (Figure S6).

### Evaluation of the effect of postbiotic-based recombinant cRANKL on adjuvanticity of oral vaccination

**Fig. 4:**
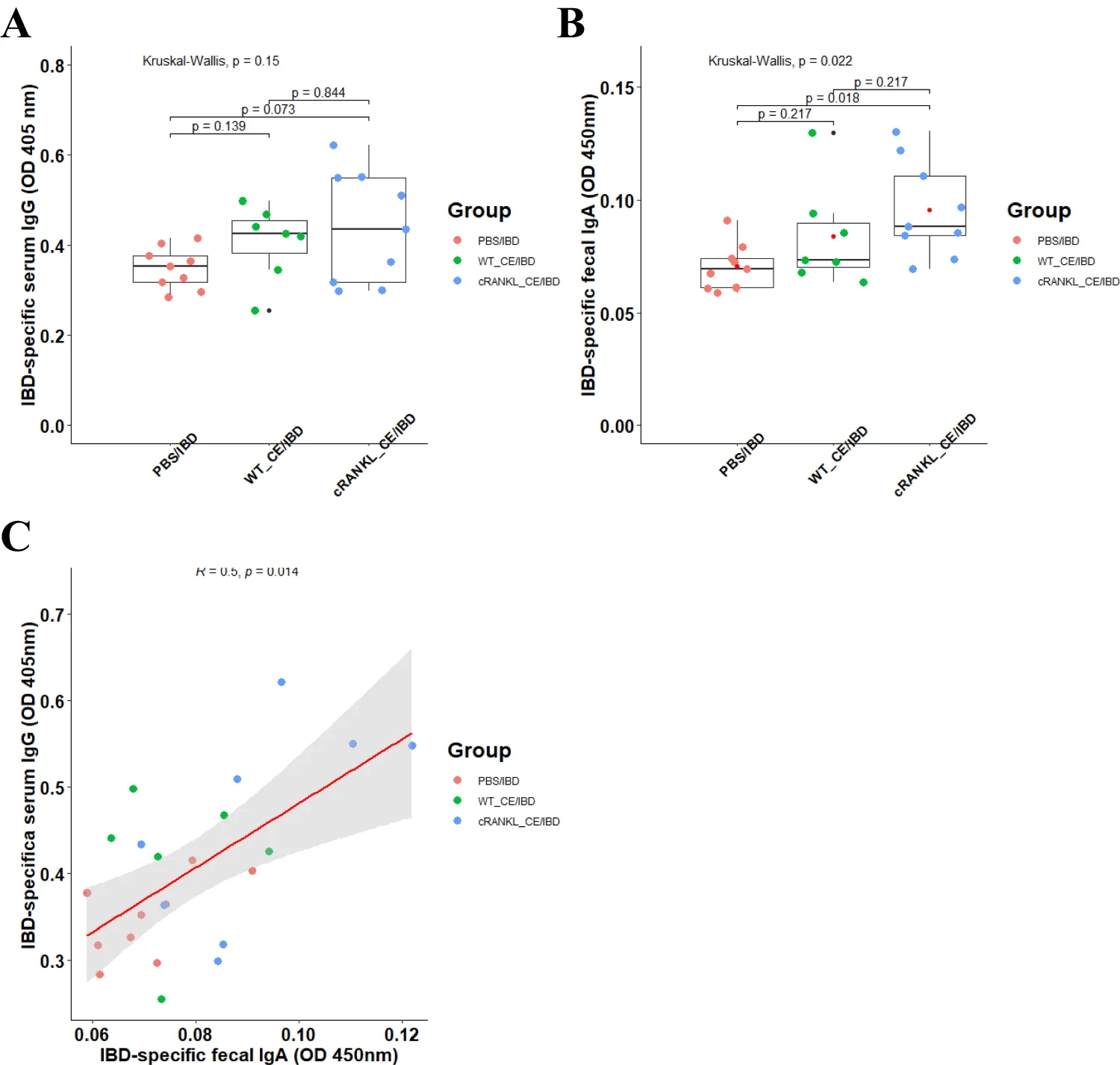
Validation of cRANKL from cell extracts as oral vaccine adjuvant in chicken (ROSS 308). **A** IBD-specific serum IgG and **B** faecal IgA. **C** The relationship between IBD-specific serum IgG and faecal IgA. Mean, red point; median, black line

To investigate whether postbiotic-based recombinant cRANKL from cell extracts enhanced IBD-specific systemic and mucosal immune responses, anti-IBD serum IgG and faecal IgA levels were measured. As shown in Fig. 4 A and B, 2 weeks after immunisation, although no significant differences were observed, the levels of serum IgG in the WT_CE/IBD (*p* = 0.139) and cRANKL_CE/IBD (*p* = 0.073) groups were higher than those in the PBS/IBD group, and no significant difference was observed between the WT_CE/IBD and cRANKL_CE/IBD groups (*p* = 0.844). This result demonstrated that cell extracts of wild-type *L. lactis* IL1403 could play a role as an adjuvant to enhance systemic immunity in chickens. The faecal IgA level of the cRANKL_CE/IBD group was significantly higher than those in the PBS/IBD group (*p* = 0.018). A positive correlation was observed between serum IgG and faecal IgA production in each individual (Fig. 4C). This result demonstrated that cell extracts with cRANKL could play a role as an adjuvant to enhance mucosal immunity in chickens.

## Discussion

Many wild-type postbiotics have been used for improving animal health owing to their effects, such as resistance to pathogens, benefits to gut barrier function, and immunomodulatory effects on the gut (Zhong et al. 2022). However, no recombinant proteins in their postbiotic form have been used in chickens.

Owing to the benefits of lactic acid bacteria (LAB) to animal health, LAB as probiotics are applicable as feed additives (Vieco-Saiz et al. 2019). In the past two decades, the application of *L. lactis* has been expanded from food additives to employment as successful microbial cell factories (Song et al. 2017). Kim et al. showed that recombinant mouse RANKL (mRANKL) secreted from recombinant *L. lactis* significantly increased the abundance of mature M cells in the mouse small intestine and improved the efficiency of oral vaccines (Kim et al. 2015). However, in this study, recombinant cRANKL was fed as a postbiotic to enhance the differentiation of M cells in the chicken small intestine. In a previous experiment, we successfully fed postbiotic-based recombinant mRANKL to mice to increase mature M cells (Xuan et al. 2022a). As non-existent organisms in the natural environment, once resealed into nature, living-modified organisms (LMOs) lead to ecological environment disorders (Prakash et al. 2011). Therefore, postbiotics can be safely used in animals. In addition, there is an advantage in that a recombinant protein purification process is not required.

In the process of producing recombinant bacteria, several strains were found to have mutations, and the transformants did not form colonies. Although the correct sequence was cloned into *L. lactis*, the gsR.h sequence appeared to be deleted or substituted, and the NS.gExR.h sequence appeared to be inserted (Figure S2). In some cases, after the translation of foreign proteins in recombinant bacterial cells, if the protein demonstrates “toxic effects” in the host, such as disruption of the normal metabolism and inhibition of cell proliferation, then bacteria will die or reduce their growth or even induce mutations to protect themselves.

**Fig. 5:**
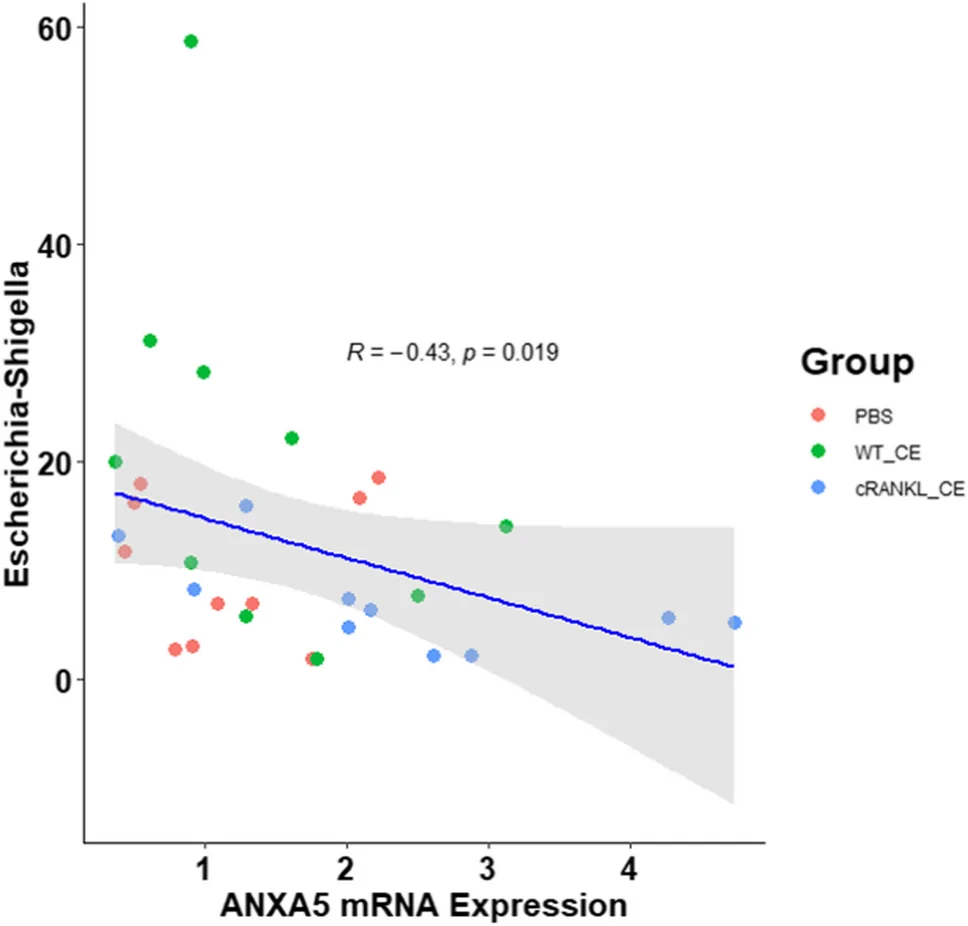
The relationship between relative abundance of *Escherichia-Shigella* and ANXA5 mRNA expression was assessed by Pearson’s correlation coefficient (*R*) and *p* value from simple linear regression

With the increase in postbiotic research, some studies have confirmed that postbiotics affect the gut microbiome (Nataraj et al. 2020; Ozma et al. 2022). Our previous study showed that cell extracts from wild-type or recombinant *L. lactis* did not critically change the mouse gut microbiome; however, in the group that was administered mRANKL contained cell extracts, the composition of the gut microbiome was similar to that of patients with rheumatoid arthritis (Xuan et al. 2022a). In this study, cell extracts derived from wild-type or recombinant strains did not significantly change the gut microbiota of chickens, and only a few genera were significantly different (Table 2). The abundance of *Lactobacillus* in the WT_CE group was higher than in the PBS and cRANKL_CE groups. In mice, cell extracts of wild-type did not affect *Lactobacillus* abundance; however, in the chicken, the abundance of *Lactobacillus* was significantly increased. Unlike in mice, the abundance of *Lactobacillus* was reduced in the group fed RANKL (Xuan et al. 2022a). This may be attributed to species-specific differences. Other reasons for the decrease in *Lactobacillus* abundance induced by cRANKL require further investigation. No significant difference was observed between groups with respect to *Escherichia-Shigella* abundance; however, the lowest abundance was found in the group fed cRANKL_CE (Table 2). ANXA5 expressed by M cells can bind to LPS of gram-negative bacteria and block endotoxin activity, indicating that ANXA5 on M cells acts as an uptake receptor for gram-negative bacteria in mice (Rand et al. 2012). The expression of *ANXA5* mRNA in the small intestine was negatively correlated with the abundance of *Escherichia-Shigella* (Fig. 5). M cells are considered to be the point of intestinal entry for antigens and invasive pathogens, such as *Shigella*, *Salmonella*, and *Yersinia* (McI Mowat et al. 2004). The abundance of *Lysinibacillus* decreased in the postbiotic groups of WT_CE and cRANKL_CE. Klaudia et al. showed that chickens infected with avian influenza had more *Lysinibacillus* in the intestine than uninfected chickens (Chrzastek et al. 2021). However, the mechanism underlying the relationship between *Lysinibacillus* and avian influenza remains unclear. In addition, the relationship between *Lysinibacillus* and immunity remains unclear. Similar to a previous study, no significant differences were observed with respect to *Lactococcus* among the three groups. This indicates that cell extracts of *L. lactis* did not elicit an immune response in the intestine (Xuan et al. 2022a, 2022b). If other strains of postbiotics were used, different changes may have been observed in the gut microbiome. In addition, no significant differences were observed in serum calcium levels among the three groups (Xuan et al. 2022a). This may indicate that recombinant cRANKL is insufficient to affect calcium metabolism in chickens.

**Table 2:** Relative abundance of phyla and genera after feeding cell extracts in chicken experiment

Taxon	Group			Kruskal–Wallis test	Dunn’s multiple comparisons test		
	PBS	WT_CE	cRANKL_CE		PBS vs. WT_CE	PBS vs. cRANKL_CE	WT_CE vs. cRANKL_CE
Phyla							
* p_Firmicutes*	74.70 ± 8.61	76.28 ± 14.92	84.27 ± 9.94	0.063	0.347	0.058	0.244
* p_Proteobacteria*	11.01 ± 7.13	20.34 ± 16.50	8.85 ± 5.99	0.107	0.267	0.461	0.112
* p_Bacteroidota*	11.34 ± 12.11	2.08 ± 3.40	5.52 ± 9.91	0.147	0.191	0.761	0.191
Genera							
* g_Lactobacillus*	14.83 ± 16.77	29.93 ± 15.62	13.69 ± 9.05	0.020*	0.023*	0.542	0.059
* g_Escherichia-Shigella*	10.28 ± 6.71	20.00 ± 16.67	7.10 ± 4.44	0.068	0.267	0.334	0.062
* g__Terrisporobacter*	1.64 ± 1.12	1.09 ± 0.88	8.99 ± 13.21	0.010*	0.322	0.071	0.009**
* g_Enterococcus*	3.62 ± 3.53	0.93 ± 0.38	2.15 ± 1.36	0.021*	0.036*	0.780	0.033*
* g__Turicibacter*	1.41 ± 0.67	1.41 ± 0.80	2.96 ± 2.25	0.011*	1.000	0.014*	0.014*
* g__Staphylococcus*	2.72 ± 3.00	0.37 ± 0.23	1.69 ± 2.86	0.015*	0.014*	0.402	0.071
* g__Tuzzerella*	0.00 ± 0.00	0.12 ± 0.39	0.13 ± 0.15	0.023*	0.525	0.025*	0.068
* g__Pseudomonas*	0.00 ± 0.00	0.00 ± 0.00	0.05 ± 0.08	0.040*	1.000	0.042*	0.042*
* g__Lysinibacillus*	0.04 ± 0.05	0.00 ± 0.00	0.02 ± 0.08	0.066	0.070	0.163	0.506
* g_Lactococcus*	0.05 ± 0.10	0.05 ± 0.05	0.07 ± 0.09	0.630	0.658	0.658	0.916

The data were expressed as the mean values ± standard deviation (SD)

The *p* values were determined using Kruskal–Wallis test (*p < 0.05; **p < 0.01; ***p < 0.001)

The Dunn’s test was used for multiple comparison between the groups (*p < 0.05; **p < 0.01; ***p < 0.001)

IBD, also known as Gumboro disease, is an immunosuppressive and highly infectious disease caused by the IBD virus (IBDV), affecting young birds and causing significant economic losses in the poultry industry worldwide (Gómez et al. 2018). Birds aged 1–14 days are less susceptible to infection and are usually protected by maternal antibodies (Mahgoub 2012). In this study, after feeding cell extracts for 12 days, chickens were immunised with an attenuated IBDV oral vaccine to test whether wild-type postbiotic or postbiotic-based recombinant cRANKL could be used as an immune adjuvant. Although no significant differences were observed, our results showed that the WT_CE/IBD and cRANKL_CE/IBD groups had higher IBD-specific serum IgG levels than the PBS/IBD group. This result indicated that cell extracts from wild-type *L. lactis* IL1403 may interfere with IgG production by enhancing intestinal immunity. The levels of IBD-specific faecal IgA were significantly higher in the cRANKL_CE/IBD group than those in the PBS/IBD group. This suggests that M cell development by cRANKL may increase the antigen transcytosis of FAE, thereby enhancing the mucosal immune response. In a previous study, when mice were fed wild-type *L. lactis* IL1403-derived cell extracts, no significant changes were observed in the small intestinal transcriptome; however, when cell extracts containing mRANKL were fed, RANKL-related genes were up- or downregulated (Xuan et al. 2022a). More indicators need to be measured to determine how wild-type cell extracts or the combination of recombinant proteins and cell extracts affect chickens.

In conclusion, we found that postbiotic-based recombinant cRANKL effectively improved M cell differentiation and the efficiency of oral vaccines. In addition, feeding postbiotic-based recombinant cRANKL did not significantly alter the gut microbiome of chickens. This strategy may be useful for the development of other functional proteins and feed additives.

## Supplementary Information

Below is the link to the electronic supplementary material.

Supplementary file1 (PDF 716 KB)

Supplementary file2 (XLSX 49 KB)

## Data Availability

Microbiome raw sequences were deposited in GenBank with the Accession Number PRJNA934978.
