# Supplementary material for: Postbiotic-based recombinant receptor activator of NF-κB ligand enhanced oral vaccine efficiency in chicken
Source: Appl Microbiol Biotechnol. 2024 Jun 26;108:397. doi: 10.1007/s00253-024-13237-9 (PMC11208263; doi:10.1007/s00253-024-13237-9)
Supplement: Supplementary file 1 [file 253_2024_13237_MOESM1_ESM.pdf]

Applied Microbiology and Biotechnology

Postbiotic-based recombinant receptor activator of NF-κB ligand enhanced oral vaccine efficiency in chicken

Biao Xuan[1], Jongbin Park[2], Seojin Choi[3], and Eun Bae Kim[3*]

[1]Department of Animal Science, College of Agriculture, Yanbian University, Yanji, 133002, China
[2]Microbiome Convergence Research Center, Korea Research Institute of Bioscience and Biotechnology (KRIBB), Daejeon 34141, South Korea
[3]Department of Applied Animal Science, College of Animal Life Sciences, Kangwon National University, Chuncheon 24341, Kangwon-do, Republic of Korea

* **Corresponding author**
Mailing address: Department of Applied Animal Science, College of Animal Life Sciences,
Kangwon National University, Chuncheon 24341, Kangwon-do, Republic of Korea.
Tel: +82-33-250-8642
Fax: +82-33-259-5574
E-mail: itanimal@kangwon.ac.kr

A.

```
gExR.h          CATATGAAGAAGAAGATCATCAGTGCAATTCTTATGTCAACCGTTATTTTATCTGCTGCC    60
gsR.h           CATATGAAGAAGAAGATCATCAGTGCAATTCTTATGTCAACCGTTATTTTATCTGCTGCC    60
                ************************************************************

gExR.h          GCTCCATTGTCTGGTGTGTATGCTGATACAGATCCATCACGTATTTCAAAAGAAGATGCT    120
gsR.h           GCTCCATTGTCTGGTGTGTATGCTGATACA------------------------------    90
                ******************************

gExR.h          CATTGTGTTCGTATGCTTTTTCGTTCACAAGAATCAATTGGTCTTCAAGATACTCCATTT    180
gsR.h           ------------------------------------------------------------    90

gExR.h          GAAAATCAAGAAGTTAAACTTATGCCAGAATCATGTCGTCGTATGAAACGTGCTCTTCAA    240
gsR.h           ------------------------------------------------------------    90

gExR.h          CGTGCTGTTCAAAAAGAAGTTCAACGTATTCTTGGTAAAGAATCACCACGTCCAGAAAAA    300
gsR.h           ---------------------------------------GAATCACCACGTCCAGAAAAA    111
                                                       *********************

gExR.h          GCTGCTATGGAAGCTATTGGTATGGAACTTTATCGTCGTAATAAACCAGAAAAACAACCA    360
gsR.h           GCTGCTATGGAAGCTATTGGTATGGAACTTTATCGTCGTAATAAACCAGAAAAACAACCA    171
                ************************************************************

gExR.h          TTTGCTCATCTTATTATTGATGATAAAAATATTCTTACTGGTACTCGTAAAGTTAATCTT    420
gsR.h           TTTGCTCATCTTATTATTGATGATAAAAATATTCTTACTGGTACTCGTAAAGTTAATCTT    231
                ************************************************************

gExR.h          ACTTCATGGCATCATAATAAAGGTCAAGCTAATCTTTCAAATATGACTTTTTCAGATGGT    480
gsR.h           ACTTCATGGCATCATAATAAAGGTCAAGCTAATCTTTCAAATATGACTTTTTCAGATGGT    291
                ************************************************************

gExR.h          AAACTTATTGTTAATCAAGATGGTTTTTATTATCTTTATGCTAATATTTGTTTTCGTCAT    540
gsR.h           AAACTTATTGTTAATCAAGATGGTTTTTATTATCTTTATGCTAATATTTGTTTTCGTCAT    351
                ************************************************************

gExR.h          CATGAAACTTCAGGTAATCTTACTAAACGTGGTCTTCAACTTATGGTTTATATGACTAAA    600
gsR.h           CATGAAACTTCAGGTAATCTTACTAAACGTGGTCTTCAACTTATGGTTTATATGACTAAA    411
                ************************************************************

gExR.h          ACTAATCTTAAAATTCGTCGTTCAGATGTTCTTATGAAAGGTGGTTCAACTAAATATTGG    660
gsR.h           ACTAATCTTAAAATTCGTCGTTCAGATGTTCTTATGAAAGGTGGTTCAACTAAATATTGG    471
                ************************************************************

gExR.h          TCAGGTAATTCAGAATTTCATTTTTATTCAGTTAATATTGGTGGTTTTCTTAAACTTAAA    720
gsR.h           TCAGGTAATTCAGAATTTCATTTTTATTCAGTTAATATTGGTGGTTTTCTTAAACTTAAA    531
                ************************************************************

gExR.h          ACTGGTGATATGATTTCAATTCAAGTTTCAAATCCACTTCTTCTTGATTCATCACAAGAA    780
gsR.h           ACTGGTGATATGATTTCAATTCAAGTTTCAAATCCACTTCTTCTTGATTCATCACAAGAA    591
                ************************************************************

gExR.h          GCTACTTATTTTGGTGCTTTTAAAGTTCGTGATCTTGATCACCATCATCACCACCATTGA    840
gsR.h           GCTACTTATTTTGGTGCTTTTAAAGTTCGTGATCTTGATCACCATCATCACCACCATTGA    651
                ************************************************************

gExR.h          CTCGAG                        846
gsR.h           CTCGAG                        657
                ******
```

B.

```
NS.gExR.h       CATATGGATCCATCACGTATTTCAAAAGAAGATGCTCATTGTGTTCGTATGCTTTTTCGT
                60
NS.gsR.h        CATATG------------------------------------------------------
                0
                ******

NS.gExR.h       TCACAAGAATCAATTGGTCTTCAAGATACTCCATTTGAAAATCAAGAAGTTAAACTTATG
                120
NS.gsR.h        ------------------------------------------------------------
                0

NS.gExR.h       CCAGAATCATGTCGTCGTATGAAACGTGCTCTTCAACGTGCTGTTCAAAAAGAAGTTCAA
                180
NS.gsR.h        ------------------------------------------------------------
                0

NS.gExR.h       CGTATTCTTGGTAAAGAATCACCACGTCCAGAAAAAGCTGCTATGGAAGCTATTGGTATG
                240
NS.gsR.h        ---------------GAATCACCACGTCCAGAAAAAGCTGCTATGGAAGCTATTGGTATG
                51
                               *********************************************

NS.gExR.h       GAACTTTATCGTCGTAATAAACCAGAAAAACAACCATTTGCTCATCTTATTATTGATGAT
                300
NS.gsR.h        GAACTTTATCGTCGTAATAAACCAGAAAAACAACCATTTGCTCATCTTATTATTGATGAT
                111
                ************************************************************

NS.gExR.h       AAAAATATTCTTACTGGTACTCGTAAAGTTAATCTTACTTCATGGCATCATAATAAAGGT
                360
NS.gsR.h        AAAAATATTCTTACTGGTACTCGTAAAGTTAATCTTACTTCATGGCATCATAATAAAGGT
                171
                ************************************************************

NS.gExR.h       CAAGCTAATCTTTCAAATATGACTTTTTCAGATGGTAAACTTATTGTTAATCAAGATGGT
                420
NS.gsR.h        CAAGCTAATCTTTCAAATATGACTTTTTCAGATGGTAAACTTATTGTTAATCAAGATGGT
                231
                ************************************************************

NS.gExR.h       TTTTATTATCTTTATGCTAATATTTGTTTTCGTCATCATGAAACTTCAGGTAATCTTACT
                480
NS.gsR.h        TTTTATTATCTTTATGCTAATATTTGTTTTCGTCATCATGAAACTTCAGGTAATCTTACT
                291
                ************************************************************

NS.gExR.h       AAACGTGGTCTTCAACTTATGGTTTATATGACTAAAACTAATCTTAAAATTCGTCGTTCA
                540
NS.gsR.h        AAACGTGGTCTTCAACTTATGGTTTATATGACTAAAACTAATCTTAAAATTCGTCGTTCA
                351
                ************************************************************

NS.gExR.h       GATGTTCTTATGAAAGGTGGTTCAACTAAATATTGGTCAGGTAATTCAGAATTTCATTTT
                600
NS.gsR.h        GATGTTCTTATGAAAGGTGGTTCAACTAAATATTGGTCAGGTAATTCAGAATTTCATTTT
                411
                ************************************************************

NS.gExR.h       TATTCAGTTAATATTGGTGGTTTTCTTAAACTTAAAACTGGTGATATGATTTCAATTCAA
                660
NS.gsR.h        TATTCAGTTAATATTGGTGGTTTTCTTAAACTTAAAACTGGTGATATGATTTCAATTCAA
                471
                ************************************************************

NS.gExR.h       GTTTCAAATCCACTTCTTCTTGATTCATCACAAGAAGCTACTTATTTTGGTGCTTTTAAA
                720
NS.gsR.h        GTTTCAAATCCACTTCTTCTTGATTCATCACAAGAAGCTACTTATTTTGGTGCTTTTAAA
                531
                ************************************************************

NS.gExR.h       GTTCGTGATCTTGATCACCATCATCACCACCATTGACTCGAG    762
NS.gsR.h        GTTCGTGATCTTGATCACCATCATCACCACCATTGACTCGAG    573
                ******************************************
```

Fig.S1 Insert sequences (A) With signal peptide (USP45) and (B) without signal peptide. gExR.h: Chicken extracellular RANKL sequence with signal peptide. gsR.h: Chicken soluble RANKL sequence with signal peptide. NS.gExR.h: Chicken extracellular RANKL sequence without signal peptide. NS.gsR.h: Chicken soluble RANKL sequence without signal peptide. Red font: Restriction sites. Green font: Signal peptide. Yellow font: His-tag.

A.

```
Synthetic_gene    ----------CATATGAAGAAGAAGATCATCAGTGCAATTCTTATGTCAACCGTTATTTT    60
pILPtuf.gsR.h     AGACATTTTTCATATG------------------------------TCAACCGTTATTTT    30
                            ******                              **************

Synthetic_gene    ATCTGCTGCCGCTCCATTGTCTGGTGTGTATGCTGATACAGAATCACCACGTCCAGAAAA    120
pILPtuf.gsR.h     ATCTGCTGCCGCTCCATTGTCTGGTGTGTATGCTGATACAGAATCACCACGTCCAGAAAA    90
                  ************************************************************

Synthetic_gene    AGCTGCTATGGAAGCTATTGGTATGGAACTTTATCGTCGTAATAAACCAGAAAAACAACC    180
pILPtuf.gsR.h     AGCTGCTATGGAAGCTATTGGTATGGAACTTTATCGTCGTAATAAACCAGAAAAACAACC    150
                  ************************************************************

Synthetic_gene    ATTTGCTCATCTTATTATTGATGATAAAAATATTCTTACTGGTACTCGTAAAGTTAATCT    240
pILPtuf.gsR.h     ATTTGCTCATCTTATTATTGATGATAAAAATATTCTTACTGGTACTCGTAAAGTTAATCT    210
                  ************************************************************

Synthetic_gene    TACTTCATGGCATCATAATAAAGGTCAAGCTAATCTTTCAAATATGACTTTTTCAGATGG    300
pILPtuf.gsR.h     TACTTCATGGCATCATAATAAAGGTCAAGCTAATCTTTCAAATATGACTTTTTCAGATGG    270
                  ************************************************************

Synthetic_gene    TAAACTTATTGTTAATCAAGATGGTTTTTATTATCTTTATGCTAATATTTGTTTTCGTCA    360
pILPtuf.gsR.h     TAAACTTATTGTTAATCAAGATGGTTTTTATTATCTTTATGCTAATATTTGTTTTCGTCA    330
                  ************************************************************

Synthetic_gene    TCATGAAACTTCAGGTAATCTTACTAAACGTGGTCTTCAACTTATGGTTTATATGACTAA    420
pILPtuf.gsR.h     TCATGAAACTTCAGGTAATCTTACTAAACGTGGTCTTCAACTTATGGTTTATATGACTAA    390
                  ************************************************************

Synthetic_gene    AACTAATCTTAAAATTCGTCGTTCAGATGTTCTTATGAAAGGTGGTTCAACTAAATATTG    480
pILPtuf.gsR.h     AACTAATCTTAAAATTCGTCGTTCAGATGTTCTTATGAAAGGTGGTTCAACTAAATATTG    450
                  ************************************************************

Synthetic_gene    GTCAGGTAATTCAGAATTTCATTTTTATTCAGTTAATATTGGTGGTTTTCTTAAACTTAA    540
pILPtuf.gsR.h     GTCAGGTAATTCAGAATTTCATTTTTATTCAGTTAATATTGGTGGTTTTCTTAAACTTAA    510
                  ************************************************************

Synthetic_gene    AACTGGTGATATGATTTCAATTCAAGTTTCAAATCCACTTCTTCTTGATTCATCACAAGA    600
pILPtuf.gsR.h     AACTGGTGATATGATTTCAATTCAAGTTTCAAATCCACTTCTTCTTGATTCATCACAAGA    570
                  ************************************************************

Synthetic_gene    AGCTACTTATTTTGGTGCTTTTAAAGTTCGTGATCTTGATCACCATCATCACCACCATTG    660
pILPtuf.gsR.h     AGCTACTTATTTTGGTGCTTTTAAAGTTCGTGATCTTGATCACCATCATCACCACCATTG    630
                  ************************************************************

Synthetic_gene    ACTCGAG----------                  677
pILPtuf.gsR.h     ACTCGAGGGATCCAGGA                  647
                  *******
```

B.

```
Synthetic_gene      ----------CATATGAAGAAGAAGATCATCAGTGCAATTCTTATGTCAACCGTTATTTT    50
pILPtuf.gsR.h       AGACATTTTTCATATGAAGAAGAAGATCATCAGTGCAATTCTTATGTCAACCGTTATTTT    60
                              **************************************************

Synthetic_gene      ATCTGCTGCCGCTCCATTGTCTGGTGTGTATGCTGATACAGAATCACCACGTCCAGAAAA    110
pILPtuf.gsR.h       ATCTGCTGCCGCTCCATTGTCTGGTGTGTATGCTGATACAGAATCACCACGTCCAGAAAA    120
                    ************************************************************

Synthetic_gene      AGCTGCTATGGAAGCTATTGGTATGGAACTTTATCGTCGTAATAAACCAGAAAAACAACC    170
pILPtuf.gsR.h       AGCTGCTATGTAAGCTATTGGTATGGAACTTTATCGTCGTAATAAACCAGAAAAACAACC    180
                    ********** *************************************************

Synthetic_gene      ATTTGCTCATCTTATTATTGATGATAAAAATATTCTTACTGGTACTCGTAAAGTTAATCT    230
pILPtuf.gsR.h       ATTTGCTCATCTTATTATTGATGATAAAAATATTCTTACTGGTACTCGTAAAGTTAATCT    240
                    ************************************************************

Synthetic_gene      TACTTCATGGCATCATAATAAAGGTCAAGCTAATCTTTCAAATATGACTTTTTCAGATGG    290
pILPtuf.gsR.h       TACTTCATGGCATCATAATAAAGGTCAAGCTAATCTTTCAAATATGACTTTTTCAGATGG    300
                    ************************************************************

Synthetic_gene      TAAACTTATTGTTAATCAAGATGGTTTTTATTATCTTTATGCTAATATTTGTTTTCGTCA    350
pILPtuf.gsR.h       TAAACTTATTGTTAATCAAGATGGTTTTTATTATCTTTATGCTAATATTTGTTTTCGTCA    360
                    ************************************************************

Synthetic_gene      TCATGAAACTTCAGGTAATCTTACTAAACGTGGTCTTCAACTTATGGTTTATATGACTAA    410
pILPtuf.gsR.h       TCATGAAACTTCAGGTAATCTTACTAAACGTGGTCTTCAACTTATGGTTTATATGACTAA    420
                    ************************************************************

Synthetic_gene      AACTAATCTTAAAATTCGTCGTTCAGATGTTCTTATGAAAGGTGGTTCAACTAAATATTG    470
pILPtuf.gsR.h       AACTAATCTTAAAATTCGTCGTTCAGATGTTCTTATGAAAGGTGGTTCAACTAAATATTG    480
                    ************************************************************

Synthetic_gene      GTCAGGTAATTCAGAATTTCATTTTTATTCAGTTAATATTGGTGGTTTTCTTAAACTTAA    530
pILPtuf.gsR.h       GTCAGGTAATTCAGAATTTCATTTTTATTCAGTTAATATTGGTGGTTTTCTTAAACTTAA    540
                    ************************************************************

Synthetic_gene      AACTGGTGATATGATTTCAATTCAAGTTTCAAATCCACTTCTTCTTGATTCATCACAAGA    590
pILPtuf.gsR.h       AACTGGTGATATGATTTCAATTCAAGTTTCAAATCCACTTCTTCTTGATTCATCACAAGA    600
                    ************************************************************

Synthetic_gene      AGCTACTTATTTTGGTGCTTTTAAAGTTCGTGATCTTGATCACCATCATCACCACCATTG    650
pILPtuf.gsR.h       AGCTACTTATTTTGGTGCTTTTAAAGTTCGTGATCTTGATCACCATCATCACCACCATTG    660
                    ************************************************************

Synthetic_gene      ACTCGAG----------                 657
pILPtuf.gsR.h       ACTCGAGGGATCCAGGA                 677
                    *******
```

C.

```
Synthetic_gene          ---------------CATATGGATCCATCACGTATTTCAAAAGAAGATGCTCATTGTGTT 45
pILPtuf.NS.gExR.h       AGACATTTTTCATAGCATATGGATCCATCACGTATTTCAAAAGAAGATGCTCATTGTGTT 60
                                       *********************************************

Synthetic_gene          CGTATGCTTTTTCGTTCACAAGAATCAATTGGTCTTCAAGATACTCCATTTGAAAATCAA 105
pILPtuf.NS.gExR.h       CGTATGCTTTTTCGTTCACAAGAATCAATTGGTCTTCAAGATACTCCATTTGAAAATCAA 120
                        ************************************************************

Synthetic_gene          GAAGTTAAACTTATGCCAGAATCATGTCGTCGTATGAAACGTGCTCTTCAACGTGCTGTT 165
pILPtuf.NS.gExR.h       GAAGTTAAACTTATGCCAGAATCATGTCGTCGTATGAAACGTGCTCTTCAACGTGCTGTT 180
                        ************************************************************

Synthetic_gene          CAAAAAGAAGTTCAACGTATTCTTGGTAAAGAATCACCACGTCCAGAAAAAGCTGCTATG 225
pILPtuf.NS.gExR.h       CAAAAAGAAGTTCAACGTATTCTTGGTAAAGAATCACCACGTCCAGAAAAAGCTGCTATG 240
                        ************************************************************

Synthetic_gene          GAAGCTATTGGTATGGAACTTTATCGTCGTAATAAACCAGAAAAACAACCATTTGCTCAT 285
pILPtuf.NS.gExR.h       GAAGCTATTGGTATGGAACTTTATCGTCGTAATAAACCAGAAAAACAACCATTTGCTCAT 300
                        ************************************************************

Synthetic_gene          CTTATTATTGATGATAAAAATATTCTTACTGGTACTCGTAAAGTTAATCTTACTTCATGG 345
pILPtuf.NS.gExR.h       CTTATTATTGATGATAAAAATATTCTTACTGGTACTCGTAAAGTTAATCTTACTTCATGG 360
                        ************************************************************

Synthetic_gene          CATCATAATAAAGGTCAAGCTAATCTTTCAAATATGACTTTTTCAGATGGTAAACTTATT 405
pILPtuf.NS.gExR.h       CATCATAATAAAGGTCAAGCTAATCTTTCAAATATGACTTTTTCAGATGGTAAACTTATT 420
                        ************************************************************

Synthetic_gene          GTTAATCAAGATGGTTTTTATTATCTTTATGCTAATATTTGTTTTCGTCATCATGAAACT 465
pILPtuf.NS.gExR.h       GTTAATCAAGATGGTTTTTATTATCTTTATGCTAATATTTGTTTTCGTCATCATGAAACT 480
                        ************************************************************

Synthetic_gene          TCAGGTAATCTTACTAAACGTGGTCTTCAACTTATGGTTTATATGACTAAAACTAATCTT 525
pILPtuf.NS.gExR.h       TCAGGTAATCTTACTAAACGTGGTCTTCAACTTATGGTTTATATGACTAAAACTAATCTT 540
                        ************************************************************

Synthetic_gene          AAAATTCGTCGTTCAGATGTTCTTATGAAAGGTGGTTCAACTAAATATTGGTCAGGTAAT 585
pILPtuf.NS.gExR.h       AAAATTCGTCGTTCAGATGTTCTTATGAAAGGTGGTTCAACTAAATATTGGTCAGGTAAT 600
                        ************************************************************

Synthetic_gene          TCAGAATTTCATTTTTATTCAGTTAATATTGGTGGTTTTCTTAAACTTAAAACTGGTGAT 645
pILPtuf.NS.gExR.h       TCAGAATTTCATTTTTATTCAGTTAATATTGGTGGTTTTCTTAAACTTAAAACTGGTGAT 660
                        ************************************************************

Synthetic_gene          ATGATTTCAATTCAAGTTTCAAATCCACTTCTTCTTGATTCATCACAAGAAGCTACTTAT 705
pILPtuf.NS.gExR.h       ATGATTTCAATTCAAGTTTCAAATCCACTTCTTCTTGATTCATCACAAGAAGCTACTTAT 720
                        ************************************************************

Synthetic_gene          TTTGGTGCTTTTAAAGTTCGTGATCTTGATCACCATCATCACCACCATTGACTCGAG--- 762
pILPtuf.NS.gExR.h       TTTGGTGCTTTTAAAGTTCGTGATCTTGATCACCATCATCACCACCATTGACTCGAGGGA 780
                        *********************************************************

Synthetic_gene          -------      762
pILPtuf.NS.gExR.h       TCCAGGA      787
```

Fig.S2 Sequencing results of deleted, substituted or inserted insert sequences. A. Deleted gsR.h. B. Substituted gsR.h. C. Inserted NS.gExR.h. Black box: Deletion, substitution or insertion. Black bold font: Vector backbone.

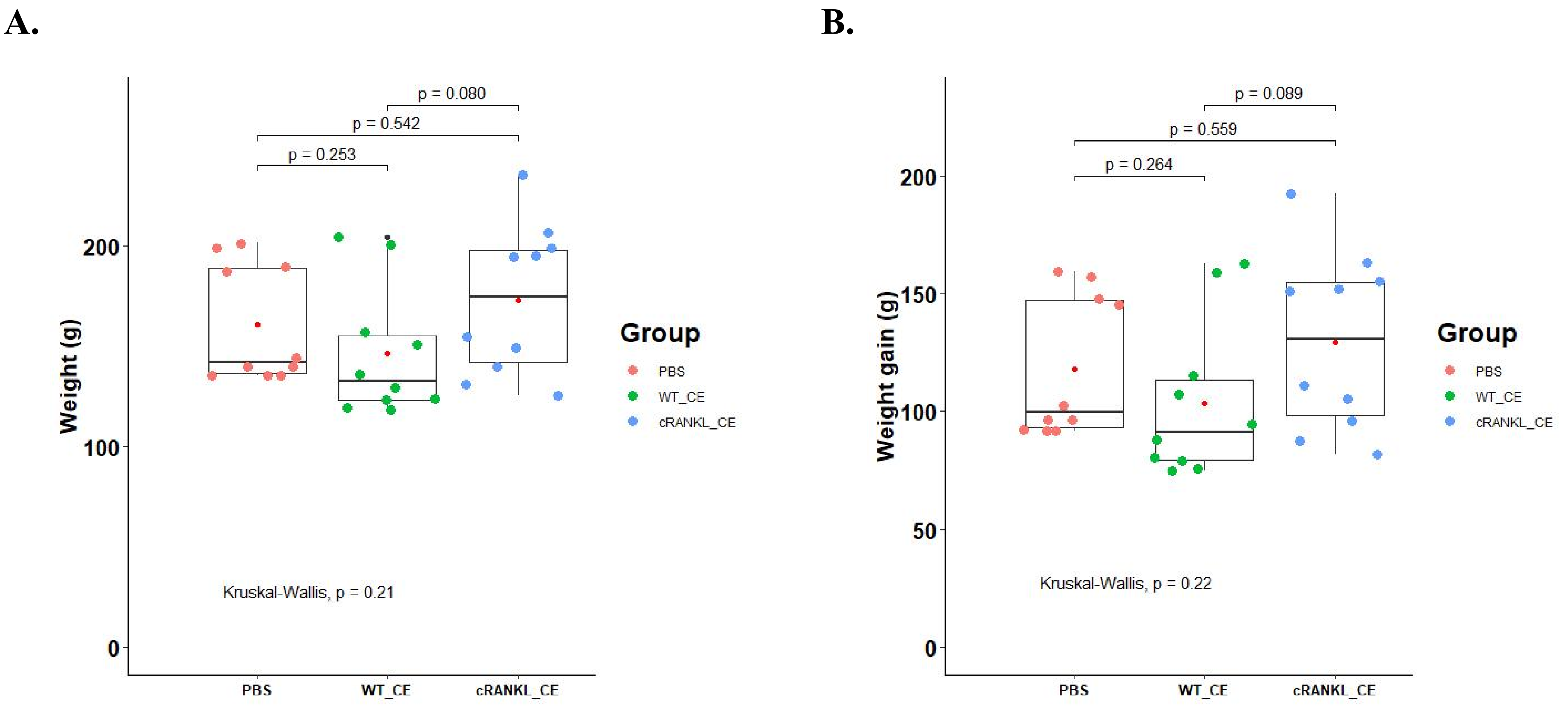

Fig.S3 Weight and weight gain after twelve consecutive days oral administration of cell extracts in chicken (ROSS 308). A. Weight (At day 13). B. Weight gain. For significance tests, Kruskal-Wallis test followed by Dunn's post-hoc test. Mean: Red point; Median: Black line.

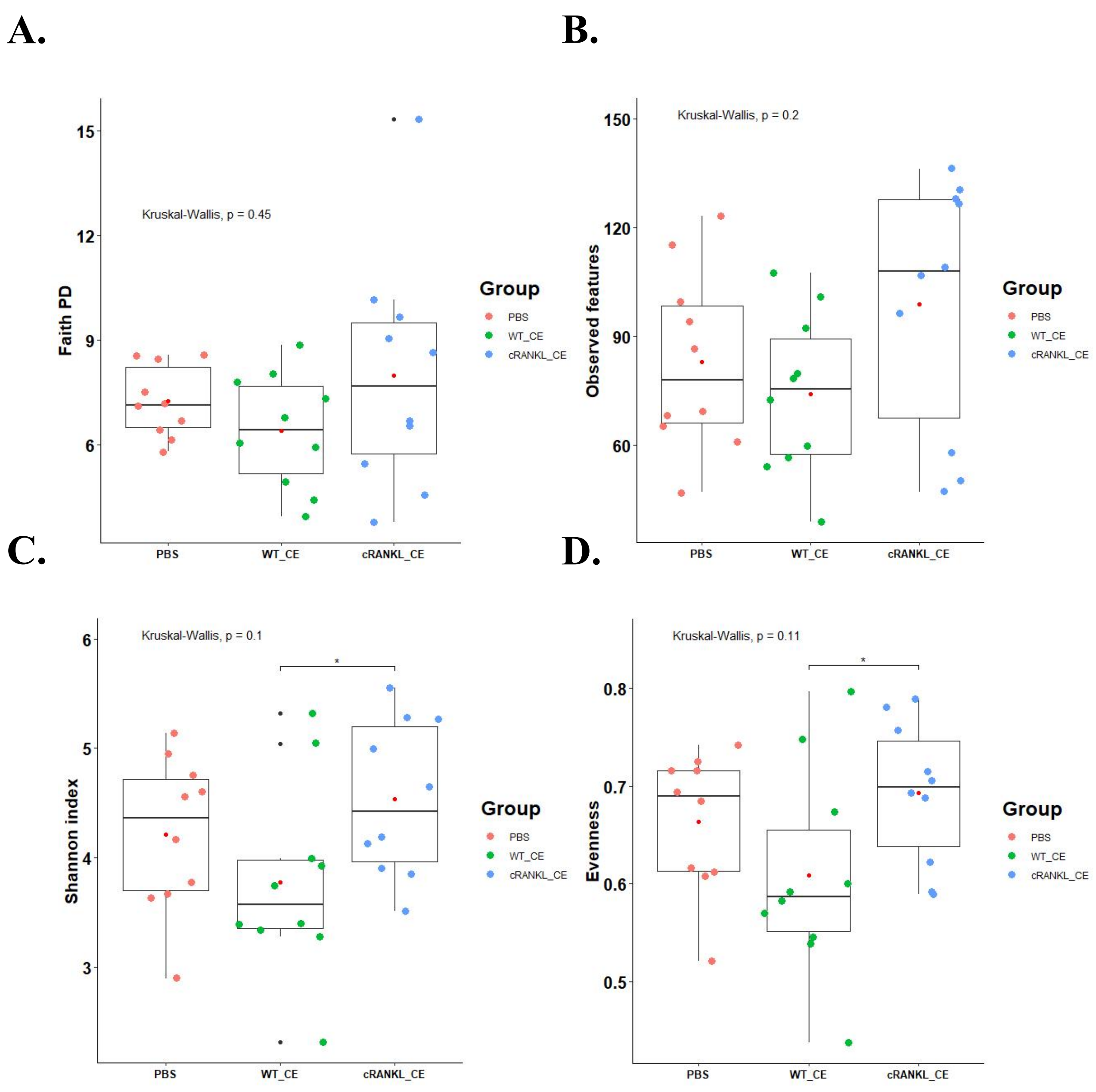

Fig.S4 Box plots of alpha-diversity indices comparing PBS, WT_CE and cRANKL_CE three groups. A. Observed features. B. Pielou evenness. C. Faith's phylogenetic diversity (Faith PD) and D. Shannon's index. *$p < 0.05$. Mean: Red point; Median: Black line.

A.

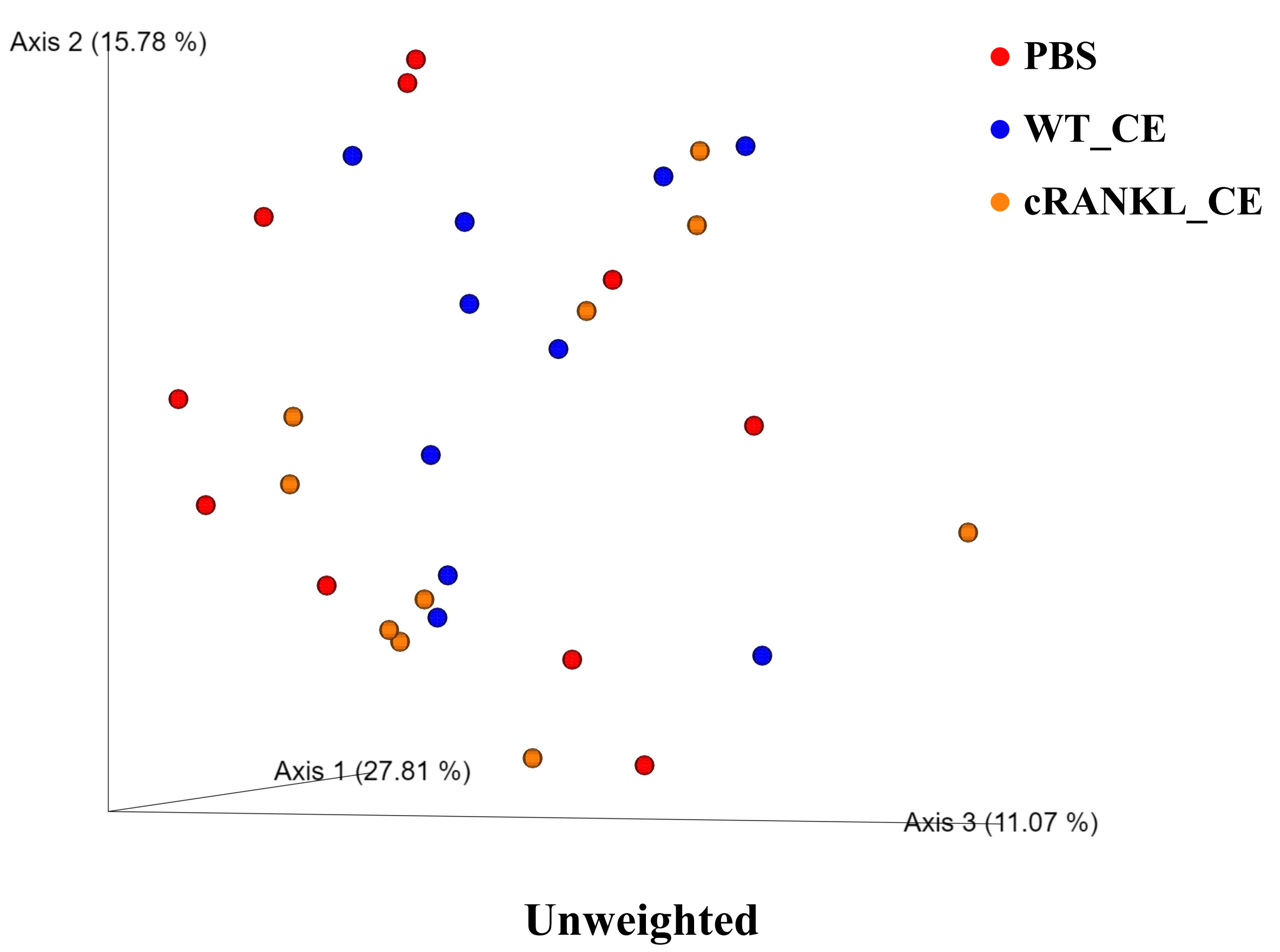

B.

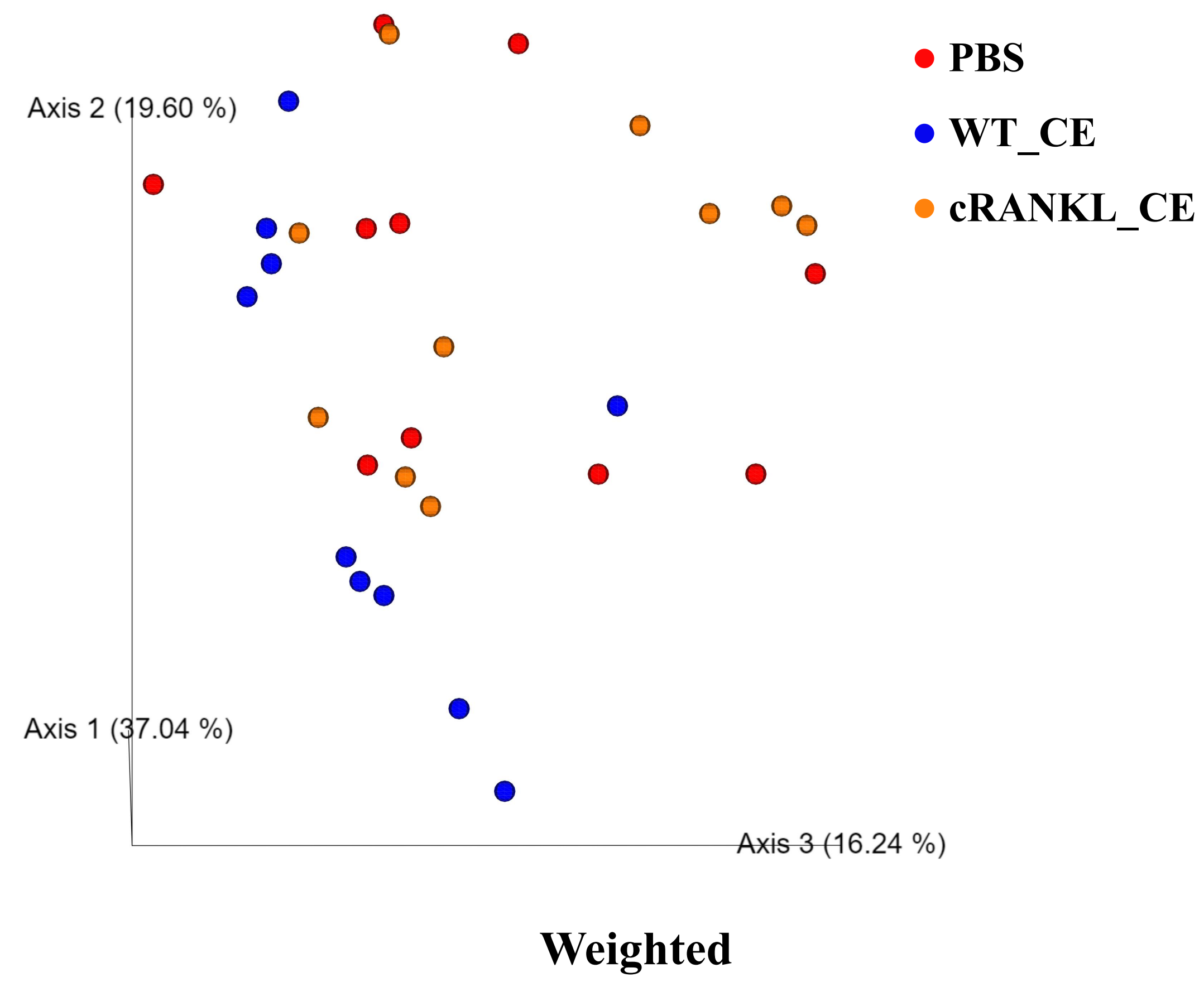

Fig.S5 Principal coordinate analysis of the microbiota among PBS, WT_CE and cRANKL_CE groups. A. Unweighted and (B) weighted based on UniFrac distances. Subject color: red, PBS (n =10); blue, WT_CE (n = 10); orange, cRANKL_CE (n = 10).

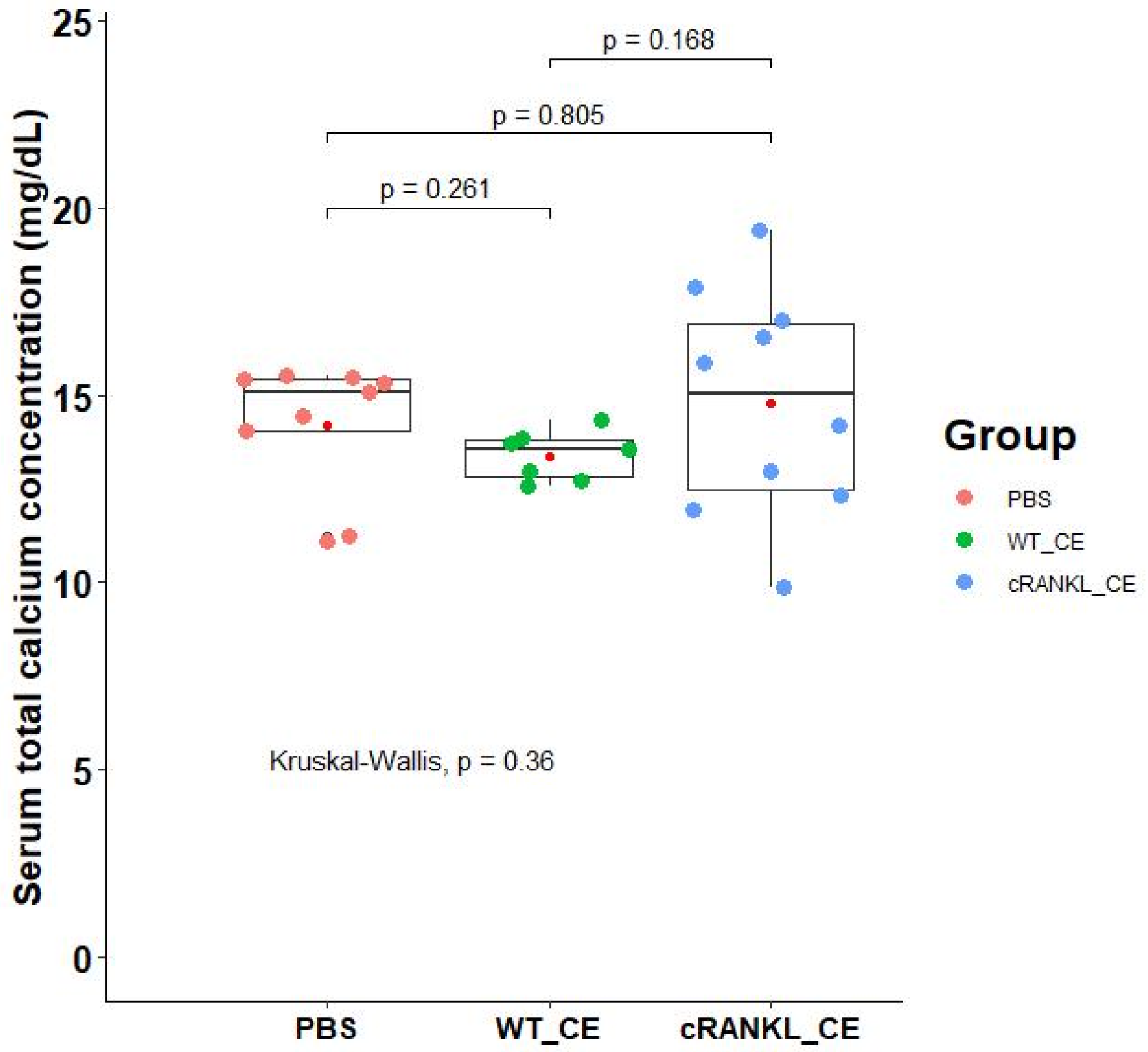

Fig.S6 Serum concentration after twelve consecutive days' oral administration of cell extracts in chicken (ROSS 308). For significance tests, Kruskal-Wallis test followed by Dunn's post-hoc test. Mean: Red point; Median: Black line.
